# Metagenome-Assembled Genome Sequences of a Biofilm Derived from Marsberg Copper Mine

**DOI:** 10.1128/MRA.01253-20

**Published:** 2021-01-14

**Authors:** Sania Arif, Heiko Nacke, Michael Hoppert

**Affiliations:** a Department of General Microbiology, Institute of Microbiology and Genetics, Georg-August-Universität, Göttingen, Germany; b Department of Genomic and Applied Microbiology, Institute of Microbiology and Genetics, Georg-August University, Göttingen, Germany; Queens College

## Abstract

We sequenced the metagenome of a biofilm collected near a leachate stream of the Marsberg copper mine (Germany) and reconstructed eight metagenome-assembled genomes. These genomes yield copper resistance through Cu(I) oxidation via multiple copper oxidases and extrusion through copper-exporting P-type ATPases.

## ANNOUNCEMENT

The historic Marsberg copper mine (51°27′12.6″N, 8°51′42.1″E) offers ambient natural conditions for the enrichment of heavy metal-resistant consortia under the influence of copper-rich (acidic) sulfidic mine waters at low temperature (10°C) (1). In February 2018, a biofilm near copper-rich leachate was aseptically collected from rock samples by using a sterile scalpel. Microbial DNA was extracted using the DNeasy PowerSoil kit (Qiagen, Venlo, Netherlands) according to the manufacturer’s protocol. The purified DNA from the biofilm sample (designated MB1) was used to generate Illumina paired-end sequencing libraries with the Nextera DNA sample preparation kit (Illumina, San Diego, CA, USA); the libraries were sequenced by employing the MiSeq reagent kit v.3 and a MiSeq instrument as stated by the manufacturer (Illumina). Default parameters were used for all software unless otherwise specified. Quality trimming of reads was performed by employing fastp v.0.20.1 (qualified quality phred score, 20; minimal read length, 50 bp) (2) and yielded 18,235,972 paired-end reads. Base correction in overlapping regions (the correction option was selected concerning fastp-based quality trimming using default parameters; this option allows identification of overlapping regions of each pair of reads, and mismatched base pairs in these regions can be corrected if one base shows high quality and the other very low quality) and removal of the automatically detected adapter sequences were performed. Low-quality bases at the 5′ and 3′ ends of reads were trimmed once the mean quality score within a sliding window of 4 dropped below 20. Sequences were *de novo* assembled into 53,638 contigs of ≥1,000 bp via metaSPAdes v.3.14.0 (3). Binning was performed using MaxBin v.2.2.7 (minimum contig length, 1,000 bp; minimum probability for binning, 0.50) (4). Application of CheckM v.1.1.2 revealed eight relatively complete metagenome-assembled genomes (MAGs) (completeness, ≥89%; contamination rate, ≤10%) (5). Each MAG was annotated using Prokka v.1.14.5 (6). Subsequently, Prokka output was analyzed by using the Pathway Tools software v.23.5 (7) with the MetaCyc database v.23.5 (8). MAGs were classified taxonomically using GTDB-Tk v.1.0.2 and the Genome Taxonomy Database (GTDB) (release 89) (9, 10).

The MAGs were classified as members of *Actinobacteria* (Mberg 009), “*Candidatus* Binatota” (Mberg 010 and 011), *Chlorobacteria* (Mberg 002, 006, 008, and 019), and *Deinococcus-Thermus* (Mberg 015). Functional analysis revealed the presence of genes for copper-sensing transcriptional repressors CsoR and RicR, copper-exporting P-type ATPases such as ActP, CptA, and CopA, and oxidation enzymes, multicopper oxidases (MCOs), involved in copper homeostasis in all MAGs. The detoxification pathways for reactive oxygen species, toxins, and antibiotic compounds involve superoxide dismutase (SOD) and peroxidases, which degrade superoxide anion radicals, and mycothiol-mediated detoxification through the enzyme Mca with thiols (11–13). All MAGs also include genes encoding aromatic compound degradation enzymes to generate ATP, which could potentially be used by copper-ATPase transporters.

### Data availability.

Raw sequencing data are available at the NCBI Sequence Read Archive (SRA) under accession number SRR12886061. The metagenome assembly is available at GenBank under accession number JADEYI000000000. The MAGs are available at GenBank under accession numbers JADMIG000000000, JADMIH000000000, JADMII000000000, JADMIJ000000000, JADMIK000000000, JADMIL000000000, JADMIM000000000, and JADMIN000000000. Prokka-based annotations of the eight MAG contigs are publicly available at the Göttingen Research Online Database (https://doi.org/10.25625/ODCARY).
